# Second-harmonic radiation by on-chip integrable mirror-symmetric nanodimers with sub-nanometric plasmonic gap

**DOI:** 10.1515/nanoph-2024-0293

**Published:** 2024-09-16

**Authors:** Junzheng Hu, Xiaofei Ye, Hui Huang, Guangxu Su, Zhekai Lv, Zhaofu Qin, Pan Hu, Fanxin Liu, Wei Wu, Peng Zhan

**Affiliations:** National Laboratory of Solid State Microstructures, and School of Physics, Nanjing University, Nanjing 210093, China; Department of Applied Physics, 12624 Zhejiang University of Technology, Hangzhou 310023, China; School of Mathematics and Physics, Guangxi Minzu University, Nanning 530006, China; Department of Electrical Engineering-Electrophysics, University of Southern California, Los Angeles, CA 90089, USA

**Keywords:** SHG, polarization symmetry-breaking, symmetric nanofinger-dimer, sub-nanometric gap, on-chip integration

## Abstract

Second-harmonic generation (SHG) facilitated by plasmonic nanostructures has drawn considerable attention, owing to its efficient frequency up-conversion at the nanoscale and potential applications in on-chip integration and nanophotonic devices. Herein, we present a nanodimer array fabricated by nanoimprinting, composed of nanofinger-pair symmetrically leaning at an off-angle with a well-defined sub-nanometric gap. Commonly, geometric symmetry would suppress the far-field SHG due to the near-field cancelling of symmetric surface SH polarization. However, we find that the light-induced surface SH polarization distribution along the wave-vector of incidence could be influenced by the off-angle, which is consistent to the requirement of SH polarization symmetry-breaking in symmetric metallic nanocavity. A dramatic enhancement of far-field SHG is achieved by tuning the off-angle of nanofinger-pair, even approaching up to over 4 orders of magnitude for an optimal value. The demonstration of SHG enhancement on our well-defined plasmonic nanodimer provides a new way of on-chip integration to activate high-efficient SH radiation, which might be potential for applications in novel nonlinear optical nanodevices with remarkable efficiency and sensitivity.

## 1 Introduction

With advancements in nanofabrication technologies and deep sub-wavelength scale light field detection capabilities, nonlinear nanophotonics has gained widespread recognition. Rapid progress has been made in various applications grounded in this discipline, including frequency conversion, imaging, biosensing, and quantum technologies [1], [2], [3], [4], [5], [6], [7]. Among them, second harmonic generation (SHG), converting two fundamental frequency photons into an SH photon, stands as a representative example of frequency up-conversion phenomena. The second-order nonlinearity in dielectric nanoparticles arises from broken centrosymmetry in the bulk crystal lattice [8], [9], [10]. For metallic nanoparticles constrained by the crystal lattice centrosymmetry in their bulk, second-order nonlinearity originates solely from the symmetry-breaking surface. Consequently, the nonlinearity strength is relatively weaker, yet the surface localization and sensitivity are enhanced, inspiring a range of exemplary studies in this domain [11], [12], [13], [14], [15], [16].

The efficiency of far-field SHG in metallic nanoparticles is largely contingent upon the geometric symmetry of the structures, as well as the degree of overlap between them and the optical near-field. Over the past decades, researchers have proposed various successful methods to enhance far-field SHG in metallic nanostructures, which primarily include: (I) breaking the geometric symmetry of nanostructure [17], [18] such as split-ring [19], L-shapes [20], T-shapes [21], [22], G-shapes [23], nanocaps [24], [25], etc., and (II) exploring novel electromagnetic mode-matching [26], [27] like magnetic mode [28], [29], [30], electric mode [31], and Fano resonance [32], [33], [34] to pursue a more potent fundamental near-field localization. Recent findings by researchers reveal that symmetry-breaking acts as a switch in the enhancement of far-field SHG in nanoparticle-mirror systems [35], [36]. Commonly, in gold nanosphere dimer with nanometer-scale gaps, surface SH polarization is already quenched in the near-field when the sizes are identical. Hence, an unresolved issue in nonlinear nanophotonics is how to effectively disrupt the symmetry of the SH polarization to enable efficient far-field SH radiation, while maintaining the highly symmetrical nanostructure. Moreover, constructing such an efficient dynamic platform that allows for nanoscale fine-tuning presents a significant challenge in the realm of nanofabrication.

**Figure 1:**
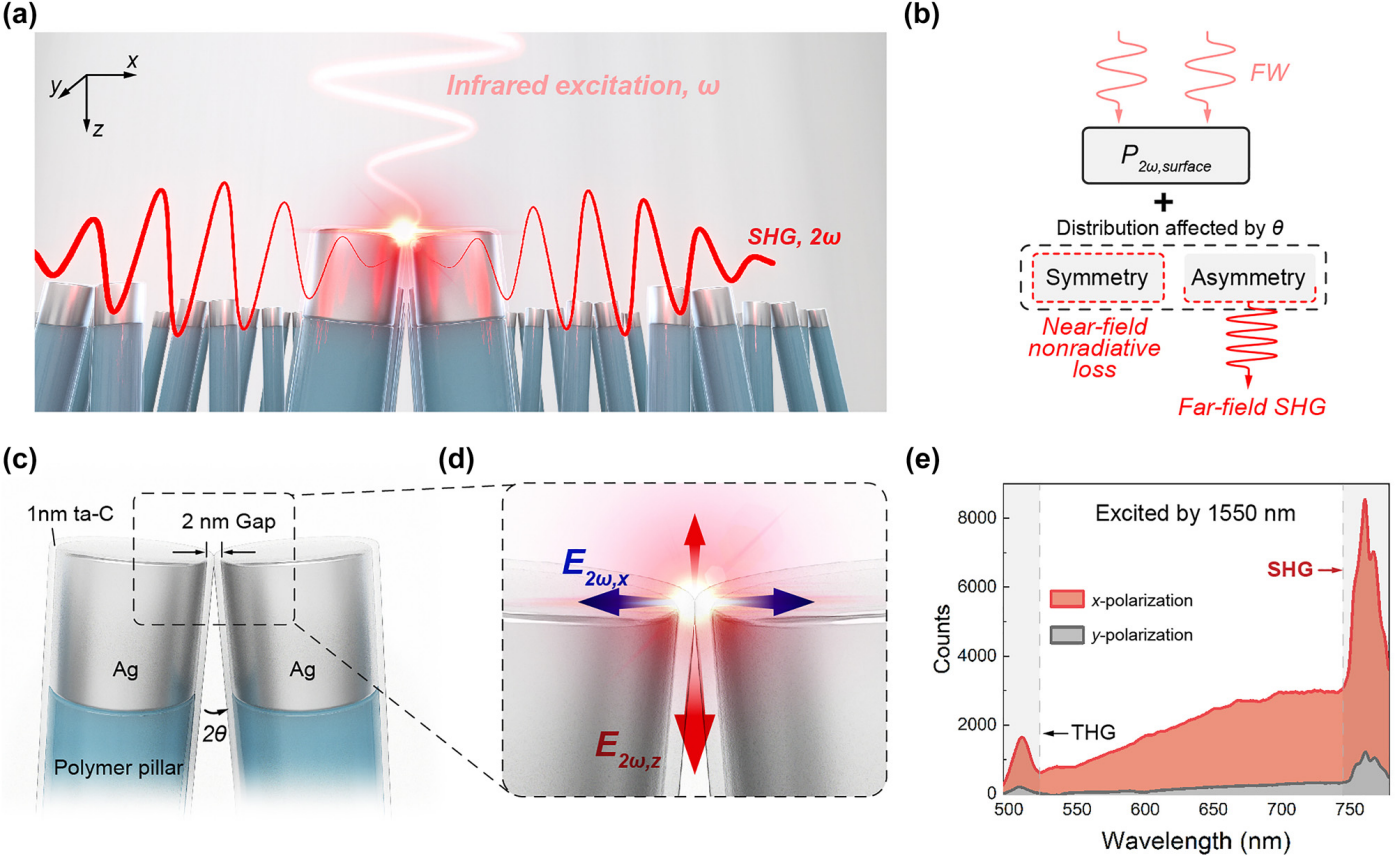
Structural description of AgND and SHG mechanism. (a) Diagram of SHG from the periodic array of AgND under the action of a *x*-polarized fundamental wave (FW). (b) SHG enhancement mechanism in metallic nanodimer, *P*_2*ω*, surface_ refers to the surface SH polarization of metallic nanostructure induced by plasmon. (c) Structural illustration of AgND. (d) The surface SH polarization distribution demonstrates an anti-bonding type in nanocavity: the *x*-component of the SH electric field distribution is symmetrical and quenched, and the *z*-component is symmetry-breaking, radiating toward the far field. (e) The nonlinear optical response spectrum excited by 1,550 nm: the FW with *x*- and *y*-polarization correspond to the excitation of AgND in bonding-dipole mode and dipole mode, respectively.

In this work, a uniform and large-area nanodimer array consisting of symmetrically leaning nanofinger-pair (i. e. a silver nanofinger dimer (AgND)) with nanogaps (Figure 1(a)) is fabricated by a modified nanoimprint lithography (NIL). Although the plasmon resonance of nanogap produces a strong surface SH polarization, the far-field SHG is highly dependent on the asymmetric distribution of SH polarization influenced by the off-angle of metallic dimer, and that reaches its maximum value for an optimal angle (Figure 1(b)). That is, while maintaining the mirror-symmetry of nanofinger-pair, we introduce an off-angle in the *z*-direction to induce asymmetry of the SH polarization distribution (Figure 1(c) and (d)). The AgND system exhibits robust far-field SHG capabilities and remarkable sensitivity in the ultraviolet-visible spectrum. Overall, our research provides a strategy for amplifying plasmonic-driven nonlinearity by building a symmetry-breaking SH polarization field through the introduction of an optimized off-angle in symmetric plasmonic systems.

## 2 Results and discussions

### 2.1 Calculation of SHG based on free-electron hydrodynamic model

In general, for metallic nanostructures, it is widely considered that only free-electrons dominate the second-order nonlinear response in the visible and near-infrared spectral range. Based on the free-electron hydrodynamic model, the SH polarization current can be expressed as [37]:

$$\begin{array}{ll} K_{NL} & =\frac{i\omega }{n_{0}e}\left[\hat{n}\frac{1}{2}\frac{3\omega +i\gamma }{2\omega +i\gamma }{\left(P_{\omega ,surface}^{⊥}\right)}^{2}\right.+\left.\hat{t}\left(P_{\omega ,surface}^{⊥}P_{\omega ,surface}^{\|}\right)\right] \end{array}$$

where *P*_*ω*, surface_ refers to the surface polarization of metallic nanostructure, the unit vectors *n* and *t* represent directions perpendicular and parallel to the metal surface, *n*_0_ denotes the free-electron density of metal, and *γ* is the electron gas collision frequency of metal. Owing to the SH field produced by plasmonic nanostructures being typically several orders of magnitude weaker than that of the pump field, we conducted simulations employing the undepleted-pump approximation. Under this approximation, the SH field is decoupled from the pump field. Additionally, due to the nonlocal effects and quantum tunneling phenomena, primarily transpiring at sub-nanometric scales [38], [39], [40], these factors were not factored into the present simulation.

### 2.2 Theoretical analysis of second harmonic generated by symmetry breaking

**Figure 2:**
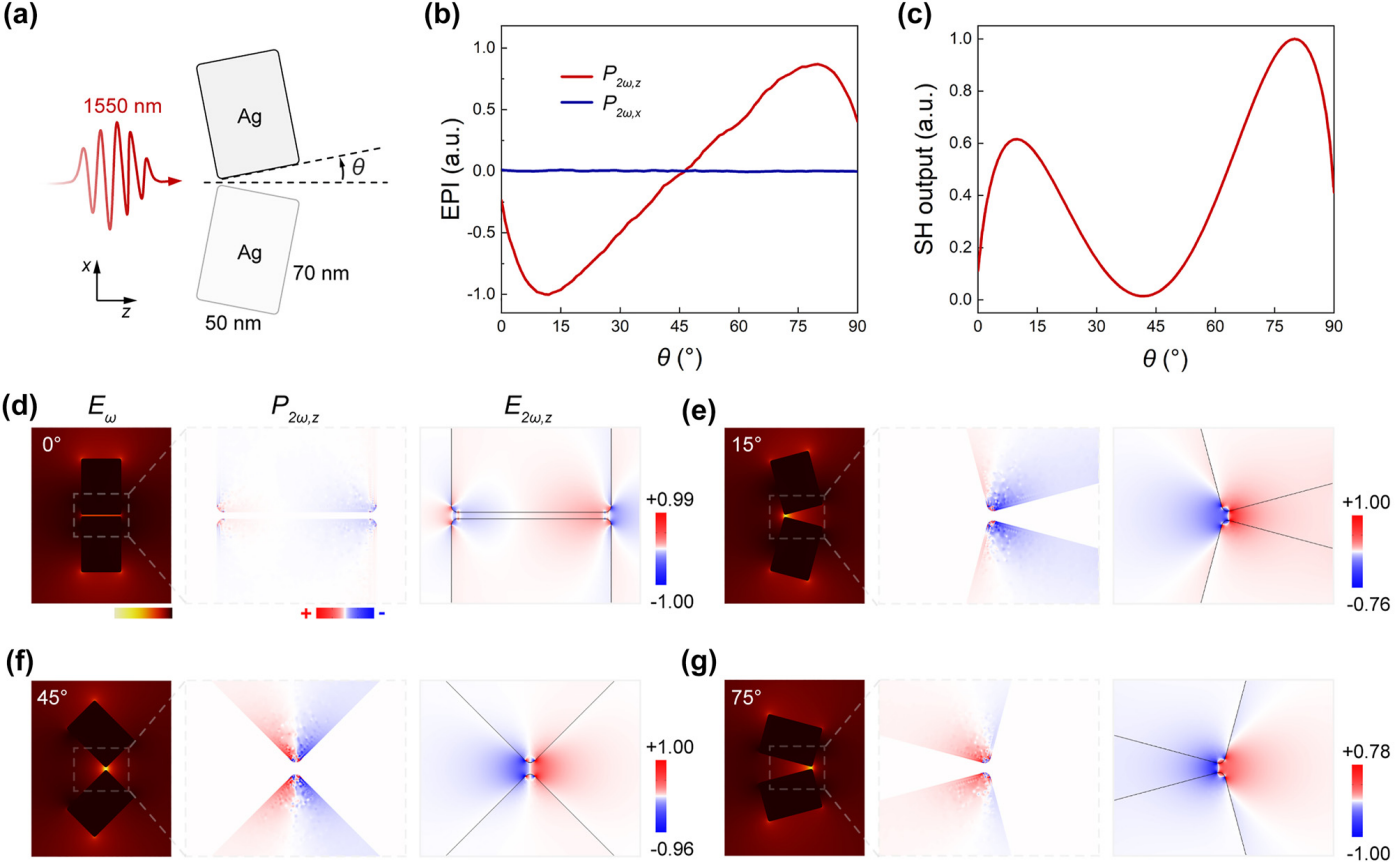
The SH origin of silver dimers with different structural asymmetries defined by the off-angle *θ*. (a) Illustration of infrared excitation of silver nanodimer. (b) Calculated surface electrical polarization integrals of nanodimer in the *x*-direction (blue), *z*-direction (red), under varying *θ*. (c) Calculated SH output from silver nanodimer at different off-angles. (d–g) Simulated near-field electric field distribution, the *z*-component of SH polarization distribution and the *z*-component of SH electric field distribution for 0°, 15°, 45°, 75° cases resulting from non-resonant plasmonic coupling.

Here a mirror-symmetric twinned silver rod-shape dimer in air is constructed, with a degree of asymmetry described by the off-angle *θ*, and a nanocavity gap of 2 nm (Figure 2(a)). The material parameters of silver are described by the Drude model [41]. To avoid the singular effects at the sharp corners during simulation, the edges of the model structure were rounded with a radius of 2 nm. Considering the rotational symmetry of the silver dimer, the simulation configuration for this part can be simplified to a 2D framework. The *x*-polarized fundamental wave (FW, 1,550 nm) excites a bonding-dipole mode in the nanodimer, while the SH polarization induced at the “hot-spot” consistently exhibits an anti-bonding type distribution. The introduced off-angle influences the far-field SHG by primarily governing the distribution of surface SH polarization (Figure 2(b) and (c)). Given the far-off plasmon resonance of the FW, the disturbance caused by plasmonic mode shift due to the change of off-angle is significantly reduced.

When the off-angle *θ* is near 45°, the SH polarization component (*P*_2*ω*, *z*_) of the silver dimer manifests a high-symmetry distribution (Figure 2(f)). The enhanced local field on the surface of the nanodimer induces strong SH polarization. However, this polarization manifests as a balanced and symmetrical distribution, and due to metal loss, this portion of energy dissipates through non-radiative processes. However, at off-angles of 15° and 75°, the SH polarization component (*P*_2*ω*, *z*_) of the nanodimer exhibits a symmetry-breaking distribution (Figure 2(e) and (g)). The SH polarized charges at the “hot-spot” generate an unbalanced SH field along the wave-vector direction, which then radiates into free space.

When the off-angle approaches 0°, the situation becomes relatively complex. Due to the light source being placed only on one side, the surface SH polarization of both ends of the nanogap presents a weak asymmetrical distribution in the *z* direction (Figure 2(d)). The far-field SH radiation presents a non-zero value and could reach a more considerable output with the matching of plasmonic resonance.

Moreover, the SH polarization component (*P*_2*ω*, *x*_) of the nanodimer always remains in a highly symmetrical state, contributing negligibly to the far-field SHG (Figures S1–2).

### 2.3 Theoretical analysis of SHG near the plasmon resonance

**Figure 3:**
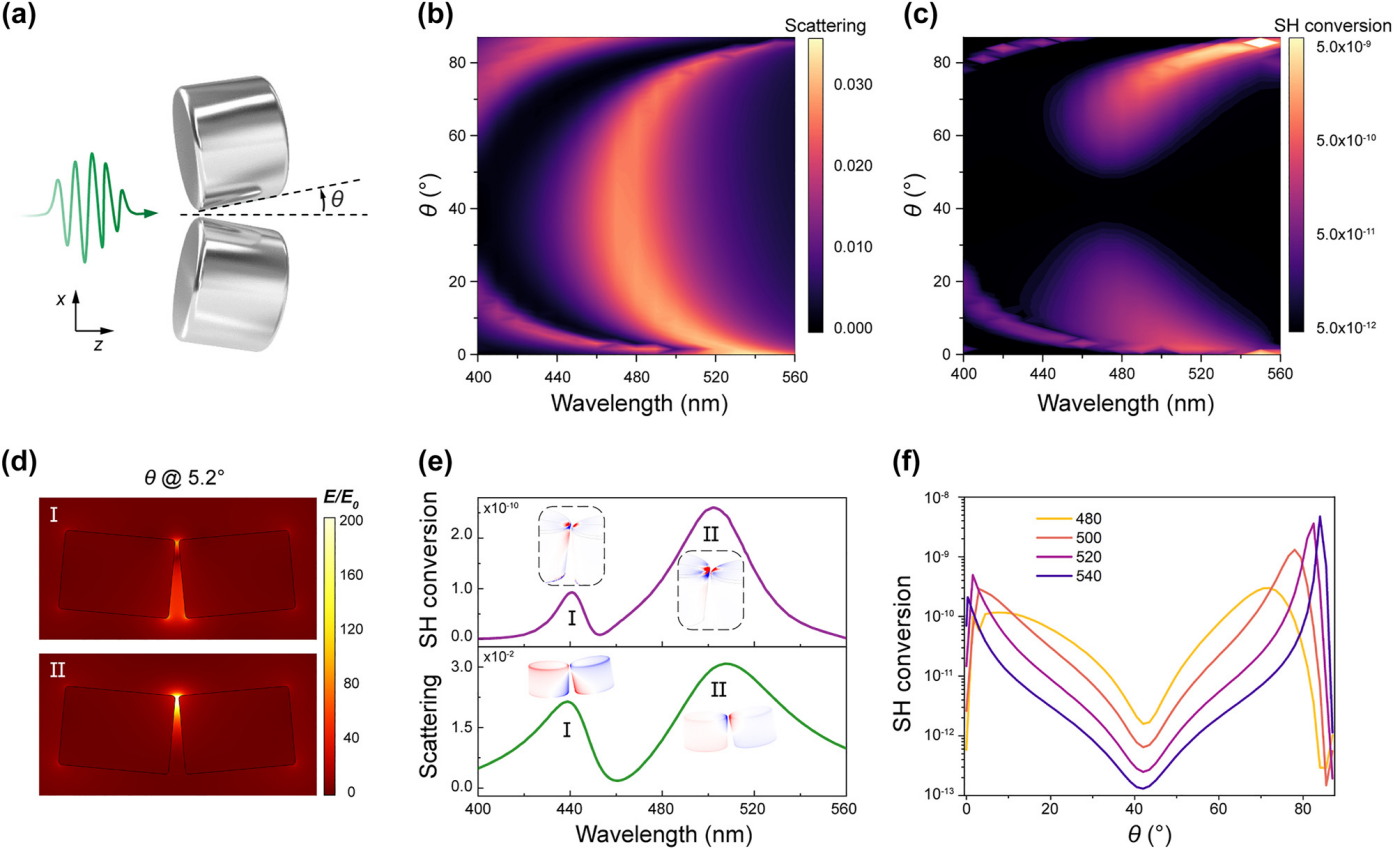
Theoretical analysis of SHG near the plasmon resonance. (a) Illustration of excitation of silver dimers near the plasmonic resonance. (b) Simulated scattering intensity versus the off-angle *θ* and wavelength of silver nanodisk dimers in a *x*-polarized beam. Mode I and II correspond to the quadrupole-like mode and bonding-dipole mode in the dimer system. (c) Simulated SH conversion versus the off-angle *θ* and wavelength of silver nanodisk dimers in a *x*-polarized beam. (d) Simulated near-field electric field distribution of mode I and II at the plasmonic resonance. (e) Simulated scattering spectrum and SH conversion of silver nanodisk dimer at the off-angle of 5.2°. (f) Simulated SH conversion versus the off-angle *θ* at different wavelength (480, 500, 520, 540 nm).

To account for the influence of the plasmon resonance alteration of AgND on the SH output during variations in the off-angle, we conducted numerical investigations. Here we studied the scattering intensity as a function of the off-angle *θ* and the wavelength of a nanodimer within an *x*-polarized beam (Figure 3(b)). Here, Mode I and Mode II correspond to a quadrupole-like mode and a dipole-bonding mode formed after the bonding of dipole modes of the nanodisk. As the off-angle of the nanodimer approaches 0°, Mode I and Mode II converge. At an off-angle of 0°, they coalesce into a mode that is predominantly governed by the bonding-dipole mechanism. By incorporating the nonlinear algorithm of the hydrodynamic surface current, we obtained simulated SH conversion versus the off-angle *θ* and wavelength of silver dimers in a *x*-polarized beam (Figure 3(c)). Notably, when the off-angle is ∼45°, a dark valley appears in the far-field SH output, due to the highly symmetrical and balanced SH polarization distribution.

Associated with AgND system, we conducted an analysis of a nanodisk dimer with an off-angle of 5.2° (Figure 3(e)). Our investigation revealed the presence of a quadrupole-like mode (Mode I) at ∼440 nm and a bonding-dipole mode (Mode II) at ∼500 nm, with the latter manifesting a superior far-field SH output conversion performance. Based on near-field electric field distribution (Figure 3(d) and (e)), it is discernible that Mode II manifests a more pronounced degree of field confinement in comparison to mode I, whereas the charge distribution of Mode I is comparatively more dispersive. Consequently, within the near-field SH polarization distribution, Mode II demonstrates a greater degree of localization. The SH polarization response elicited by Mode I is comparatively subdued, resulting in a far-field SH output that is approximately an order of magnitude inferior in amplitude to that of Mode II.

Under the combined influence of structural asymmetry and plasmonic resonance modes, the far-field SH output of the nanodimer exhibits extreme sensitivity to changes in the off-angle within the spectral range of 480–540 nm. By tuning the off-angle of nanodimer, the far-field SHG can be enhanced by an additional 4 orders of magnitude. Under single-wavelength excitation (such as 540 nm), even a small off-angle change within a range of 5° near the peak would lead to up to 2 orders of magnitude changes in the far-field SH output (Figure 3(f)). As the off-angle approaches 45°, the SH polarization distribution reaches a highly symmetrical state, where minor angular variations have little influence on the overall far-field SH output. These observational results have laid a foundation for the development of highly sensitive detectors based on SHG from metallic nanostructures.

### 2.4 Experimental observation of highly-efficient SH radiation from AgND

To exemplify the aforementioned model in experiments, we designed a mirror-symmetric twinned AgND, which is composed of a high aspect ratio polymer pillar, silver nanodisks, and a uniformly coated 1 nm ta-C (tetrahedral amorphous carbon) film. And the ta-C film is notable for its compactness and small positive electron affinity [42]. Through immersion in a highly volatile solvent, the neighboring flexible ta-C-capped-nanofingers collapse onto each other through capillary forces, forming a physical-contacted dimer array with a ∼5°-off-angle of and a 2 nm-nanogap determined by sub-nano ta-C layer. Notably, precise control of the height of the polymer pillars during the etching process give a promising way for altering the off-angle, albeit it is still difficult under our current experimental conditions.

Under the non-resonant excitation (1,550 nm), the AgND demonstrates typical nonlinear optical spectra (Figure 1(e)). The FW of *x*- and *y*-polarizations corresponds to the excitation of bonding-dipole mode and dipole mode respectively. SHG and THG, as well as their broadband feature, are observed in the spectra. The broadband feature is attributed to the electron scattering of the second-order and third-order mode of the nanodimer and the irregular surface [43], [44]. Notably, at the same excitation intensity, the nonlinear response obtained from *x*-polarization excitation is nearly an order of magnitude higher than that obtained from *y*-polarization excitation. This can be attributed to the fact that the bonding-dipole mode excited by the FW parallel to the dimer axis can generate a stronger “hotspot” at nanocavity.

**Figure 4:**
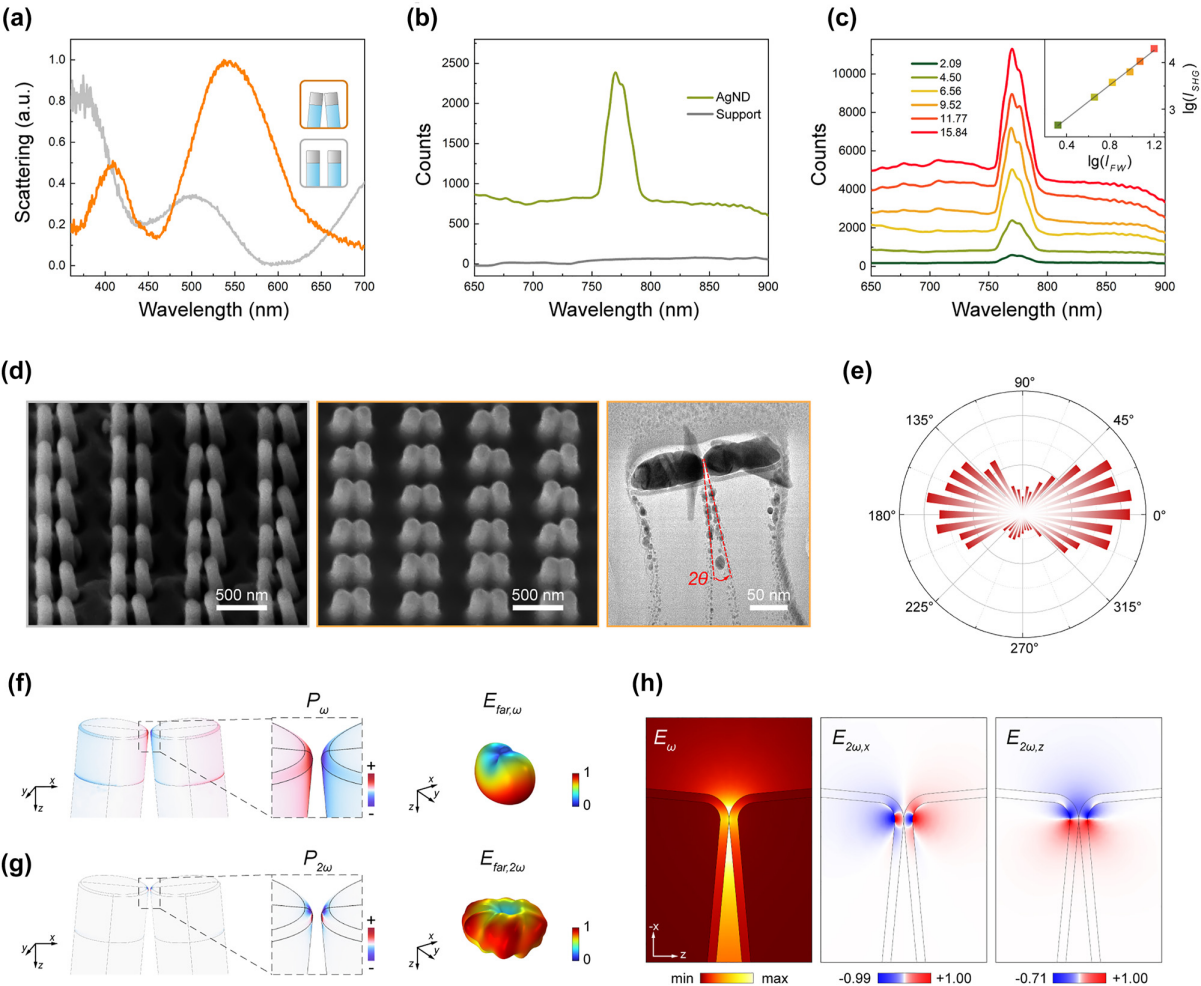
Experimental observation of SHG from AgND and numerical simulation. (a) Scattering spectrum prior to the collapse (gray) and after the collapse (yellow) for the AgND. (b) SH spectrum of the experiment: AgND (green), ta-C support (gray). (c) SH spectrum measured on non-resonant excitation with 1,550 nm at different excitation intensities. The inset shows a logarithmic linear fit of the SH peak intensity as a function of the FW power, with a slope of 1.88. (d) SEM image prior to the collapse and after the collapse for the AgND, scale bar 500 nm; TEM cross-sectional image of AgND after collapse, scale bar 50 nm. (e) Polar plot of the SH intensity at different excitation polarization angles. (f) Polarization distributions and far-field radiation result from the non-resonant plasmon of AgND. (g) SH polarization distributions and far-field SH radiation result from the surface nonlinear sources of AgND. (h) Simulated electric field distribution of AgND at the FW of 1,550 nm; the distribution of the normalized SH electric field in the *x*- and *z*-component.

According to the scattering spectrum (Figure 4(a)), the collapsed AgND has two localized surface plasmon resonance (LSPR) bands centered ∼540 nm (bonding-dipole mode) and ∼413 nm (quadrupole-like mode), respectively. The structure before collapse only has one LSPR band centered ∼360 nm (dipole mode). Under non-resonant excitation, a pronounced SH signal is observed on the AgND, while no SH signal is detected on the substrate solely covered by ta-C and silver layers (Figure 4(b)). Thus, the nonlinear response originating from ta-C can be disregarded. The SH spectral response is measured at varying excitation power intensities (Figure 4(c)). The inset illustrates a linear relationship between the logarithmic peak photon number of SH and the logarithmic energy of the fundamental wave, with a slope of 1.88. This verifies that the signals collected are indeed SH. Notably, the SHG conversion efficiency here reaches 2.01 × 10^–10^ W^−1^, approaching that of dual-resonance plasmonic nanostructures [26]. Furthermore, by using a half-wave plate to control the polarization angle of the fundamental wave, the AgND generates a strong polarization-dependent SH signal. This indicates a significant consistency with the polarization related to theoretical plasmonic hot-spots (Figure 4(e)).

The FW with *x*-polarization provokes a bonding-dipole mode of the AgND (Figure S4), consequently instigating plasmonic oscillation along the *x*-axis and generating far-field radiation. Throughout the frequency up-conversion process, the induced SH polarization on the metallic surface predominantly presents an antibonding type. The optimal off-angle dictates the asymmetric distribution of SH polarization along the *z*-axis. In the wave-vector direction (along the *z*-axis), the SH polarization field exhibits a symmetry-breaking distribution, denoted as *E*_2*ω*, *z*_, characterized by a discrepancy of approximately 30 % in intensity between the positive (*z*+) and negative (*z*−) directions. Meanwhile, the SH polarization distribution demonstrates almost perfect symmetry along the *x* direction. Consequently, the total SH polarization oscillates along the *z*-axis, coupling into the dark mode channel and radiating SH to the far-field (Figure 4(f)–(h)). In addition, while the far-field radiation of the AgND is influenced by the presence of polymer pillars and ta-C film, its fundamental radiation properties are essentially unchanged, as demonstrated by the silver nanodisk dimers (Figure S6).

Furthermore, our fabrication process, including electron beam lithography, nanoimprint lithography, and reactive ion etching, is compatible with standard chip manufacturing processes, enhancing their potential for integration with existing photonic circuits. Additionally, these approaches offer advantages such as high resolution, low cost, high throughput, and environmental friendliness. The subsequent integration can significantly enhance device performance by improving light manipulation at the nanoscale, which is essential for the development of highly efficient, miniaturized photonic devices.

## 3 Conclusions

Theoretically, we have investigated the far-field SHG influenced by light-induced electromagnetic asymmetry in the highly mirror-symmetric twinned metallic nanodimer. Experimentally, through the Nanoimprint Lithography (NIL) method, we constructed mirror-symmetric plasmonic nanostructures with an extremely narrow gap and an off-angle. By optimizing the off-angle, we successfully broke the optical symmetry of surface SH polarization along the wave-vector direction in the nanocavity, thereby facilitating high-quality far-field SHG. Even under non-resonant excitation, the SH conversion efficiency from AgND is comparable to those of dual-resonance plasmonic nanostructures. The peak output intensity of the SH exhibits significant exponential sensitivity to the off-angle near the resonance position, primarily due to the interaction between the bonding-dipole mode, quadrupole-like mode, and the introduced asymmetry of the off-angle. Moreover, the NIL method employed in our approach could potentially lead to the fabrication of more complicated plasmonic structures, such as multilayer metal-dielectric (MMD) cavities [29], [30] with sub-nanometric gaps. This advancement would depend on further improvements in controlling the collapsing angle of nano-pillar pairs and overcoming the unforeseen challenges in fabricating multilayer stacks. According to experimental and theoretical results, these nanostructures possess great potential in spectral conversion from extreme ultraviolet to visible light, high-sensitivity photodetectors, and quantum devices.

## 4 Methods

### 4.1 Fabrication of silver nanofinger dimer arrays deflected at small angles

Polymer nanofingers are synthesized through a combination of sophisticated techniques, including electron beam lithography (EBL), reactive ion etching (RIE), and nanoimprint lithography (NIL), with typical diameters and heights of 70 nm and 650 nm, respectively. The lattice parameters and gap sizes between adjacent nanofingers are 500 nm and 130 nm, respectively. Subsequently, a 50 nm thick Ag layer is deposited on the top of the nanofingers via electron beam evaporation. Afterwards, a 1 nm ta-C film is uniformly deposited on the Ag nanofingers using filtered cathodic vacuum arc technology. The sample is tilted at a 45-degree angle and rotated to achieve better coverage. After drying in a high-volatility solvent, capillary forces induce the formation of a physically contacting dimer system with an approximate 5° off-angle in the vertical direction and a uniform gap size. By adjusting the height of the polymer pillars during the resist etching process, or the spacing between adjacent pillars in the design of the daughter mold, the off-angle can be controlled within a certain range. Figure 4(d) presents scanning electron microscope (SEM) and transmission electron microscope (TEM) images of the nanofinger array before and after collapse, which exhibit good agreement with the design. For more detailed information on the synthesis of polymer nanofingers, please refer to our previous work [42], [45].

## Supplementary Material

Supplementary Material Details
