# Supplementary material for: Second-harmonic radiation by on-chip integrable mirror-symmetric nanodimers with sub-nanometric plasmonic gap
Source: Nanophotonics. 2024 Sep 16;14(11):1907–15. doi: 10.1515/nanoph-2024-0293 (PMC12133245; doi:10.1515/nanoph-2024-0293)

Supporting Information

Second-Harmonic Radiation by On-chip Integrable Mirror-symmetric Nanodimers with Sub-nanometric Plasmonic Gap

Junzheng Hu^1†^, Xiaofei Ye^1†^, Hui Huang^1†^, Guangxu Su^2†^, Zhekai Lv^2^, Zhaofu Qin^3^, Pan Hu^4^, Fanxin Liu^2*^, Peng Zhan^1*^, Wei Wu^4*^

*1National Laboratory of Solid State Microstructures, and School of Physics, Nanjing University, Nanjing, P. R. China, 210093*

*2Department of Applied Physics, Zhejiang University of Technology, Hangzhou, P. R. China, 310023*

*3School of Mathematics and Physics, Guangxi Minzu University, Nanning, P. R. China, 530006*

*4Ming Hsieh Department of Electrical and Computer Engineering, University of Southern California, California, Los Angeles, USA, 90007*

E-mails: liufanxin@zjut.edu.cn, zhanpeng@nju.edu.cn, wu.w@usc.edu

**S1. Numerical FEM calculations**

The simulation was carried out in the frequency domain solver using a commercial finite element solver, COMSOL Multiphysics, under the undepleted-pump approximation. In the simulation, the fundamental wave-vector was oriented along the +z direction, while the polarization of the incident light and the axis of the nanodimer were along the x-axis. We numerically simulated the near-field and SH polarization field of the nanodimer. The nonlinear process was decomposed into two linear scattering processes: first, the fundamental field was solved in the first frequency domain solver, and then the SH polarization source current on the surface of the metal nanoparticle was computed using the second frequency domain solver. In addition, the far-field radiation of nanodimer was obtained by performing a near-field to far-field transformation of the simulated field at the SH wavelength.

It is noteworthy to consider non-local effects of electrons and quantum tunneling occurring only in narrower nanoparticle gaps (sub-nanometer scale). Due to the tunneling barrier height of 2.76 eV, the Ag/ta-C/Ag system adopts classical electromagnetic field theory for simulation.

**S2. Distribution of surface SH polarization in the x direction**

Under the non-resonant excitation of the x-polarized beam, the second harmonic (SH) polarization of the nanodimer demonstrates an anti-bonding type distribution. Here, the surface polarization of individual nanodisks (N1 or N2) within the dimer system has been integrated. Notably, during the process of off-angle variation, the EPI of both nanodisks consistently maintains a high degree of symmetry with respect to zero (Fig.S1), and the distribution of the x-component of the SH polarization and the SH electric field likewise persist in a highly symmetrical and balanced state (Fig.S2). Consequently, in the x-direction, the SH polarization induced by plasmons contributes negligibly to the far-field SHG.


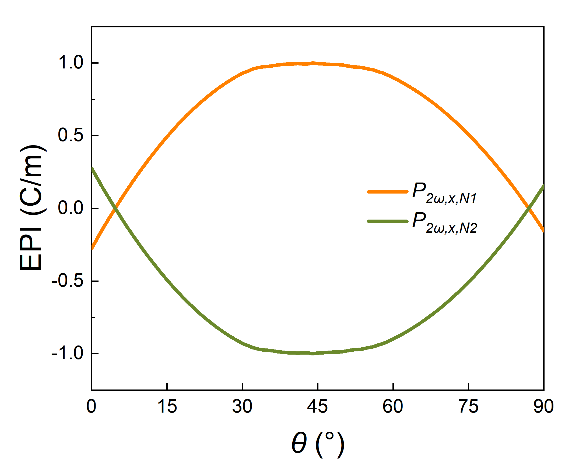


**Figure S1.** Calculated surface electrical polarization integrals of single nanodisk (N1 or N2) in the x-direction, under varying θ.


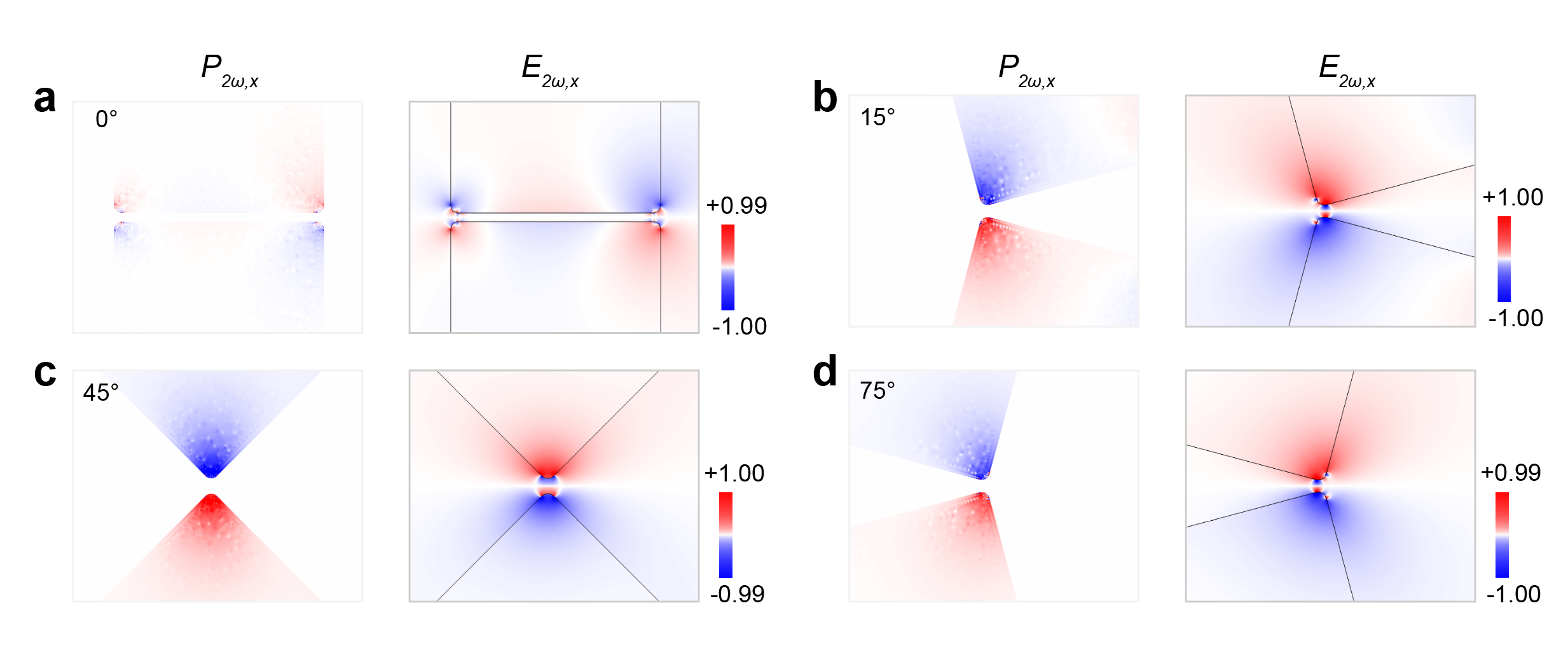


**Figure S2.** Simulated x-component of SH polarization distribution and x-component of SH electric field distribution for 0°, 15°, 45°, 75° cases resulting from non-resonant plasmonic coupling.

**S3. Experimental setup for nonlinear optical spectroscopy**

In our experimental setup, a femtosecond laser characterized by a repetition frequency of 50 MHz and pulse width less than 0.1 ps is utilized to induce SHG from the array of AgND. The FW polarization direction, i.e., the electric field vector, aligns along the axis of the dimer. The excitation light was transmitted through a fixed wavelength of 1550 nm, and its intensity was modulated by a dynamically adjustable attenuator (Fig.S3). The polarization of light was controlled by a half-wave plate, and the light was directed to the sample through a microscope system. The harmonic signal was acquired using a 100× objective with a numerical aperture (NA) of 0.95, and the FW component was filtered out using a bandpass filter. Ultimately, the signal is directed either to a CCD imaging port or spectrometer collection port by a mirror selector. A laser power detector is located between the half-wave plate and the lens, installed at a position that allows for rotation.

Notably, the focal spot diameter, determined by the size of the Airy spot in the objective focal plane, is estimated to be ~1.99 μm. The pitch of the silver nanofinger array is 500 nm. Given the shape and size of the focal spot, it can be estimate that a dozen dimers are excited during the measurements.


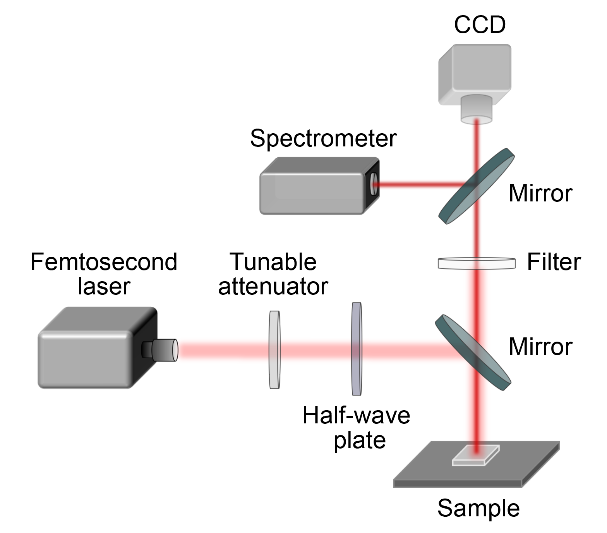


**Figure S3.** Experimental setup for nonlinear optical spectroscopy.

**S4. Scattering spectra and multipolar decompositions**

In an endeavor to delve deeper into the electromagnetic characteristics of silver nanodisk dimer with an off-angle of 5.2°, we utilized multipole expansions of EM scattering to ascertain the contribution of electric dipole (ED), magnetic dipole (MD), electric quadrupole (EQ), and magnetic quadrupole (MQ) to the overall field. The research findings revealed that near LSPR, the contribution of the ED is predominant. More specifically, the two peaks in the red-line segment correspond respectively to the quadrupole-like mode and the bonding-dipole mode in the dimer system, while the contributions from the MD and EQ are relatively minor, and the contribution from the MQ can be essentially disregarded (Fig.S4. a). In the regions away from the LSPR, the electric dipole's contribution remains dominant, predominantly manifesting as the bonding-dipole mode within the dimer system, with other components' contributions being practically negligible (Fig.S4. b).


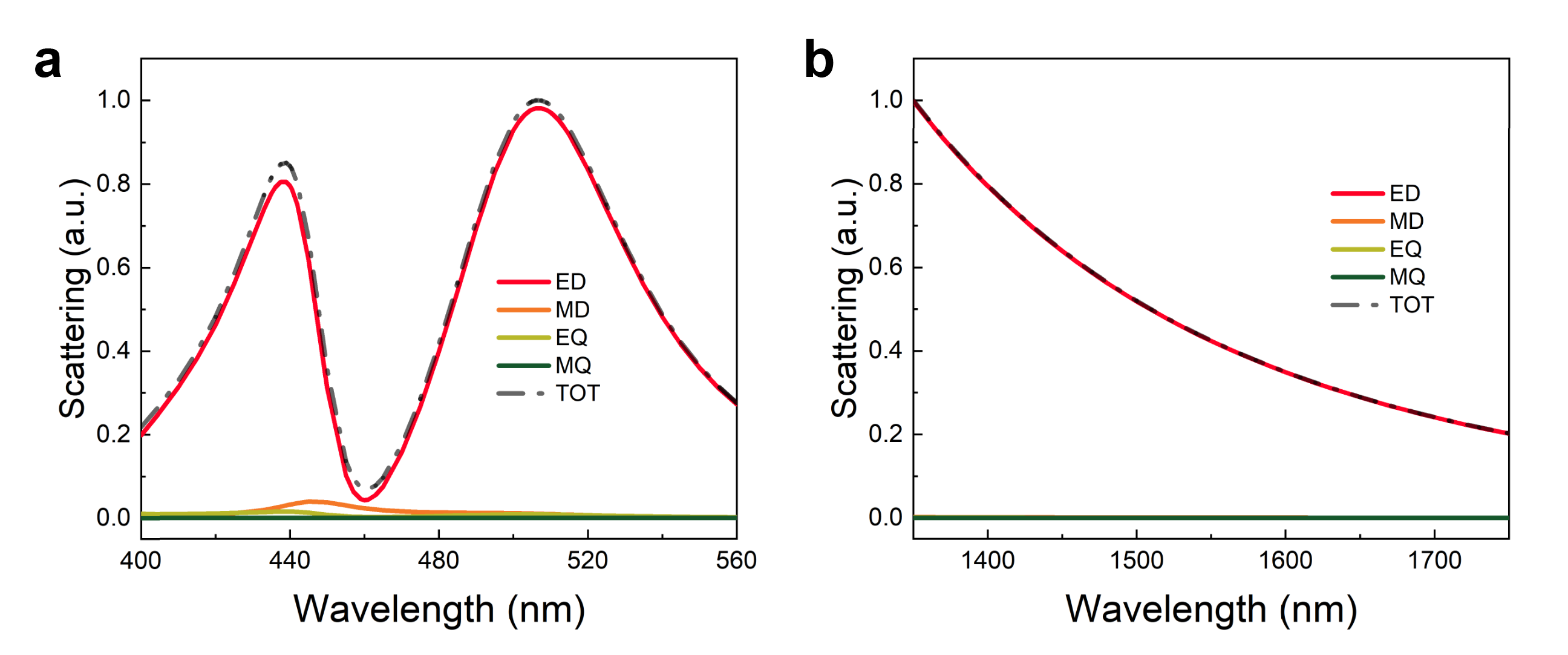


**Figure S4.** Scattering spectra and multipolar decompositions of silver nanodisk dimer near resonance (a) and away from resonance (b).

**S5. Numerical analysis of individual nanodisk at the plasmon resonance**


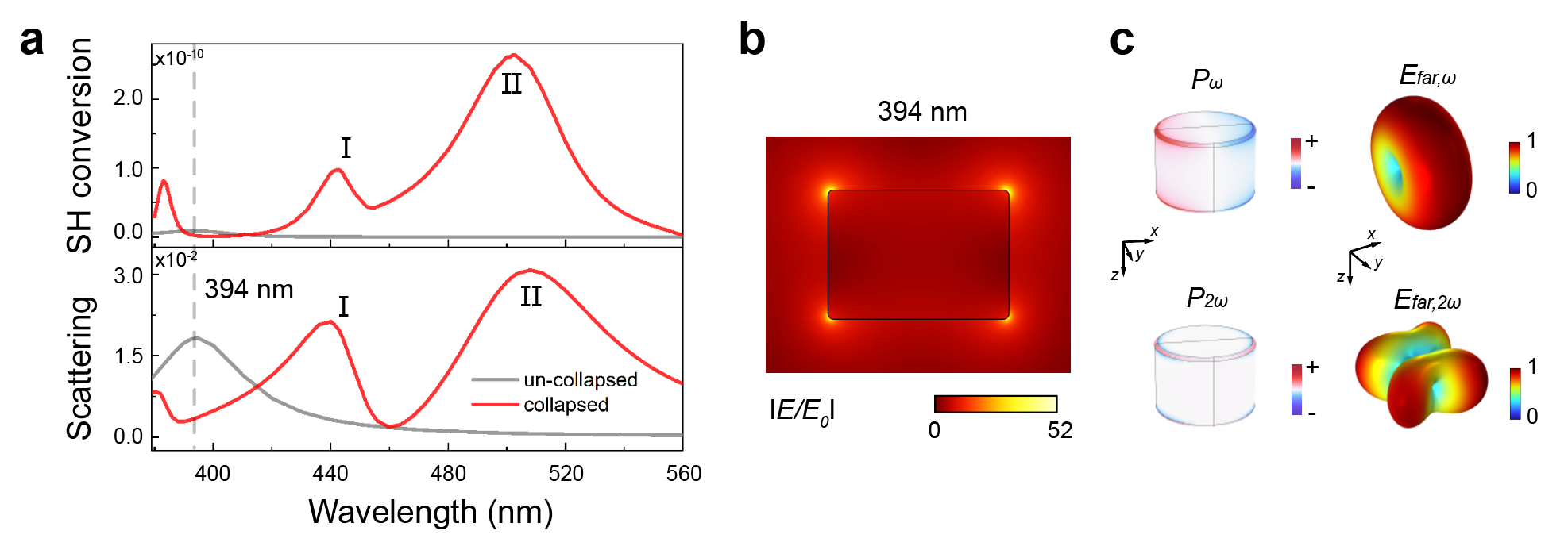


**Figure S5.** (a) Simulated scattering spectrum and SH conversion of a nanodisk dimer and individual nanodisk. The off-angle of the nanodisk dimer is 5.2°. (b) Simulated near-field electric field distribution of individual nanodisk at dipole resonance. (c) Polarization distributions, SH polarization distributions, far-field radiation and far-field SH radiation result from the dipole resonance of individual nanodisk.

**S6. Numerical near-field distribution and far-field radiation of silver nanodisk dimer**


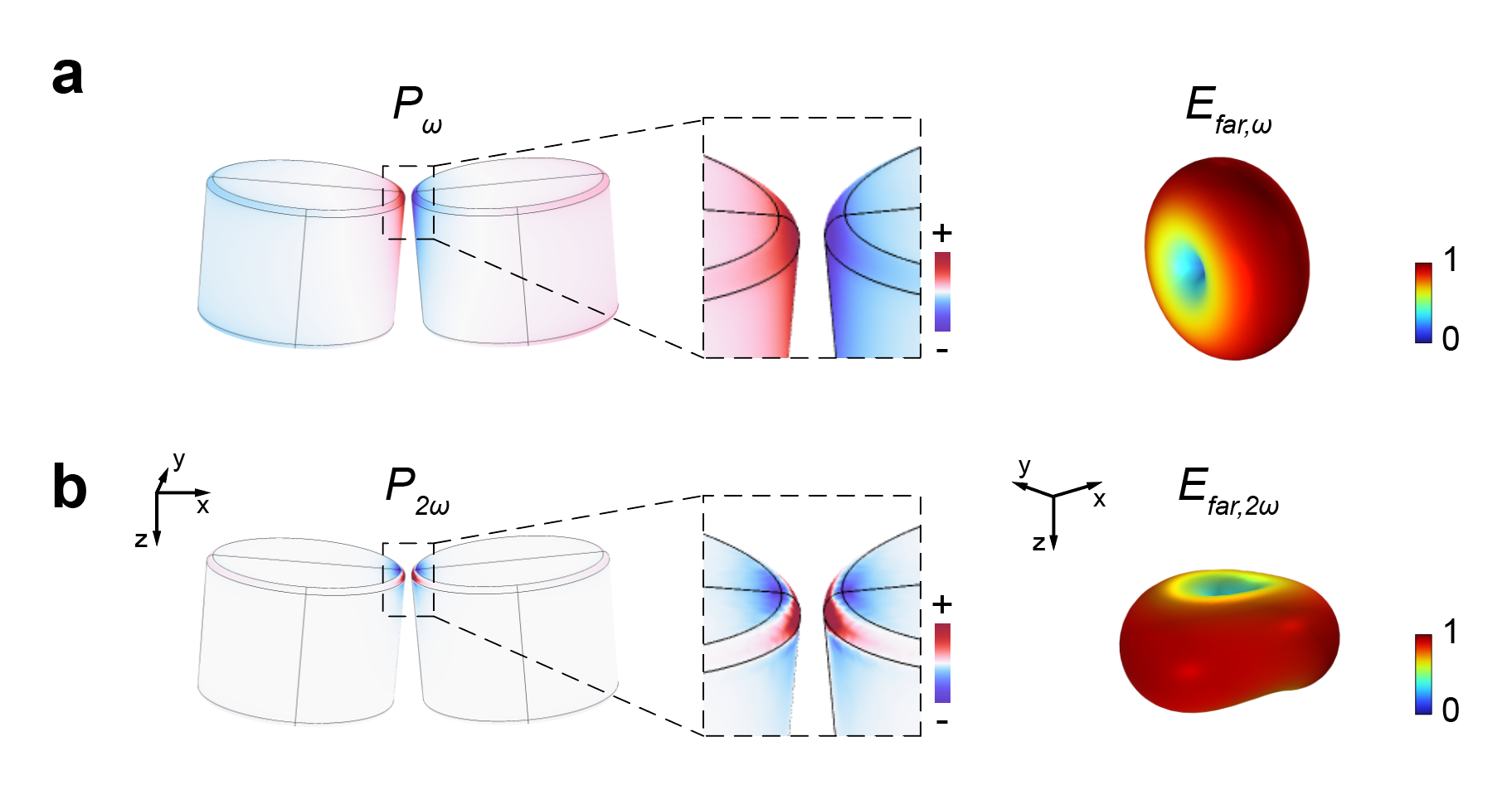


**Figure S6.** (a) Polarization distributions and far-field radiation result from the non-resonant plasmon of silver nanodisk dimer. (b) SH polarization distributions and far-field SH radiation result from the surface nonlinear sources of silver nanodisk dimer.

**Table S1. Parameters of experimental setups.**
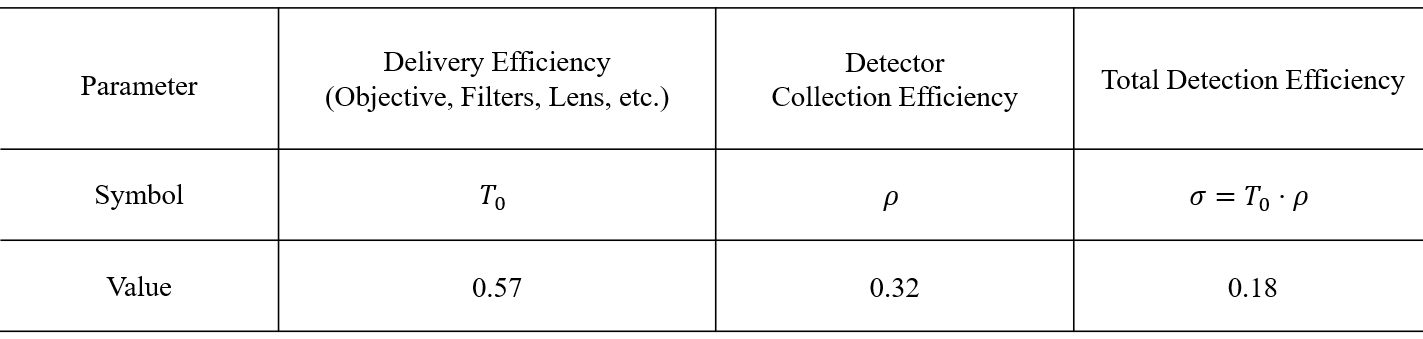


**Table S2. Evaluation of the second-harmonic conversion efficiency**


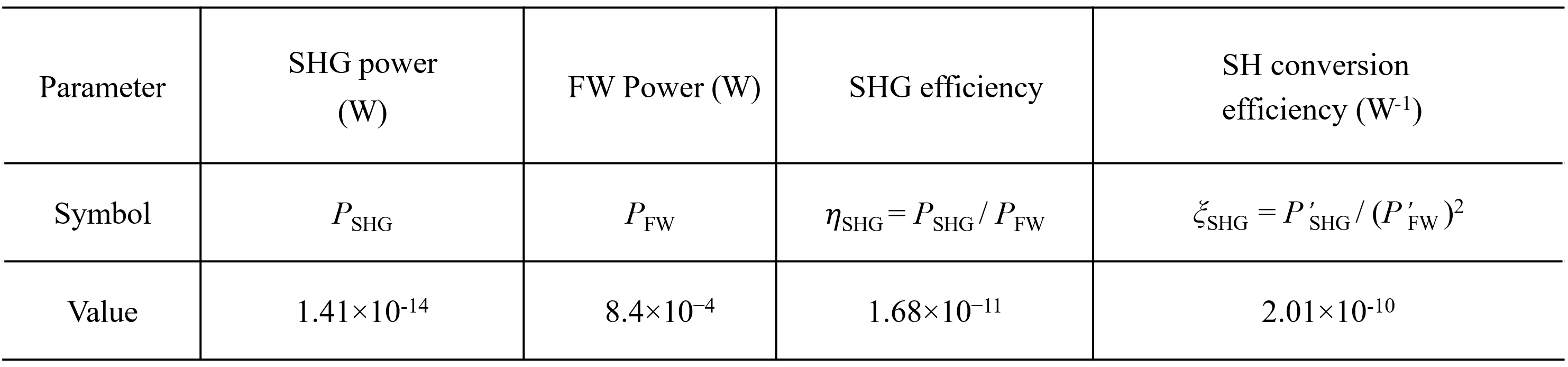

Supplement: Supplementary file 1 — Supplementary Material Details [file j_nanoph-2024-0293_suppl_001.docx]
